# Rapid predictive simulations with complex musculoskeletal models suggest that diverse healthy and pathological human gaits can emerge from similar control strategies

**DOI:** 10.1098/rsif.2019.0402

**Published:** 2019-08-21

**Authors:** Antoine Falisse, Gil Serrancolí, Christopher L. Dembia, Joris Gillis, Ilse Jonkers, Friedl De Groote

**Affiliations:** 1 Department of Movement Sciences, KU Leuven, Leuven, Belgium; 2 Department of Mechanical Engineering, KU Leuven, Leuven, Belgium; 3 Department of Mechanical Engineering, Universitat Politècnica de Catalunya, Barcelona, Catalunya, Spain; 4 Department of Mechanical Engineering, Stanford University, Stanford, CA, USA; 5 DMMS Lab, Flanders Make, Leuven, Belgium

**Keywords:** biomechanics, locomotion, optimal control, three-dimensional

## Abstract

Physics-based predictive simulations of human movement have the potential to support personalized medicine, but large computational costs and difficulties to model control strategies have limited their use. We have developed a computationally efficient optimal control framework to predict human gaits based on optimization of a performance criterion without relying on experimental data. The framework generates three-dimensional muscle-driven simulations in 36 min on average—more than 20 times faster than existing simulations—by using direct collocation, implicit differential equations and algorithmic differentiation. Using this framework, we identified a multi-objective performance criterion combining energy and effort considerations that produces physiologically realistic walking gaits. The same criterion also predicted the walk-to-run transition and clinical gait deficiencies caused by muscle weakness and prosthesis use, suggesting that diverse healthy and pathological gaits can emerge from the same control strategy. The ability to predict the mechanics and energetics of a broad range of gaits with complex three-dimensional musculoskeletal models will allow testing novel hypotheses about gait control and hasten the development of optimal treatments for neuro-musculoskeletal disorders.

## 1. Introduction

Scientists have long tried to decipher the principles underlying bipedal locomotion with the aim of improving human gait performance and treatment of neuro-musculoskeletal disorders. A powerful approach to this problem is the use of physics-based predictive simulations that generate *de novo* movements based on a mathematical description of the neuro-musculoskeletal system without relying on experimental data. Such simulations can explore diverse hypotheses about mechanisms underlying locomotion that are difficult to study through experiments. The high computational time of predictive simulations has favoured the use of conceptual models that only describe the most prominent features of the musculoskeletal system. Predictive simulations based on conceptual models have contributed to our understanding of the mechanics [1,2] and energetics [3–6] of bipedal locomotion. However, such models provide limited support for personalized clinical decision-making, since they do not sufficiently describe the musculoskeletal structures and motor control processes underlying gait that may be affected by treatment. An orthopaedic surgeon considering a rectus femoris transfer in a patient with cerebral palsy cannot predict the effect of the surgery on the walking pattern of the patient using conceptual models. By contrast, complex musculoskeletal models that include the many degrees of freedom of the skeleton and the many muscles actuating the lower limbs have the potential to make such predictions. Yet these complex models are computationally expensive in predictive simulations [7–10] and, therefore, the field has not explored their ability to predict the broad range of gaits encountered under different environments, pathologies and augmentations. Such generalizability is a prerequisite for using predictive simulations to design optimal treatments.

Predictive simulations typically optimize a performance criterion that describes the high-level goal of the motor task without relying on experimental motion data. Yet it remains unclear what such a criterion would be for human gait. Experimental studies suggest that humans select gait features, such as step frequency and length, that optimize the cost of transport (COT, defined as metabolic energy consumed per unit distance travelled) [11] and that they continuously optimize the COT during walking [12]. Similarly, energy considerations have been suggested to drive the walk-to-run transition as gait speed increases [13]. Following these experimental observations, numerous simulation studies have used energy-based performance criteria to predict human walking or running [5–10]. Predictive simulations based on conceptual models also showed that the same energy-based criterion produced walking at low speeds and running at high speeds [5,6]. However, criteria centred on muscle activity, used as surrogates for muscle effort and fatigue, have also been suggested to underlie gait [14–16]. Simulation studies based on two-dimensional musculoskeletal models reported that using a performance criterion based on muscle activity better predicted the preferred walking speed in elderly [16] and resulted in more accurate kinematics during running [15] as compared to using an energy-based performance criterion. Yet it is unclear whether these observations hold for simulations based on three-dimensional models. Further, gait might be governed by multiple performance criteria [15,17] but the effect of combining different criteria on the predicted gait pattern has not been widely explored with complex three-dimensional models, likely due to the associated computational costs.

Finally, it remains unclear whether a single task-level performance criterion can explain the range of gaits adopted by humans in different contexts. No simulation study has yet explored whether different gaits, such as healthy walking and running or pathological gaits, can emerge from the same underlying control strategy when using complex three-dimensional models.

The purpose of our study was threefold. First, we developed a computationally efficient optimal control framework to predict human gaits based on complex musculoskeletal models. To this aim, we combined direct collocation, implicit differential equations and algorithmic differentiation. Second, we sought a performance criterion that could accurately predict human walking. To this aim, we explored a wide range of walking-related performance criteria and selected the criteria that best described walking at a self-selected speed. Third, we tested whether our framework could predict healthy and pathological gaits when altering gait speed and musculoskeletal properties but without altering the control strategy (i.e. using the same performance criterion). To this aim, we simulated gait (i) at different speeds, (ii) with muscle strength deficits and (iii) with a lower leg prosthesis and compared our simulation results to those from experiments. The computational efficiency of our framework (goal 1) allowed us to explore potential cost functions (goal 2) and to test the ability to predict the mechanics and energetics of a range of human gaits based on complex musculoskeletal models (goal 3).

## 2. Material and methods

### 2.1. Musculoskeletal model

We used an OpenSim musculoskeletal model with 29 d.f. (6 between pelvis and ground; 3, 1, and 2 at each hip, knee and ankle, respectively; 3 at the lumbar joint between trunk and pelvis; and 4 per arm), 92 muscles actuating the lower limbs and trunk (43 per leg and six actuating the lumbar joint), eight ideal torque actuators at the arms and six contact spheres per foot [18,19]. To increase computational speed, we fixed the moving knee flexion axis to its anatomical reference position; moving and fixed knee flexion axes give similar results for gait [20]. We added passive stiffness (exponential) and damping (linear) to the joints of the lower limbs and trunk to model ligaments and other passive structures [7].

We used Raasch's model [21,22] to describe muscle excitation–activation coupling and a Hill-type muscle model [23,24] to describe muscle–tendon interaction and the dependence of muscle force on fibre length and velocity. We modelled skeletal motion with Newtonian rigid body dynamics and compliant Hunt–Crossley foot-ground contacts [19,25]. We used smooth approximations of the Hunt–Crossley model that were twice continuously differentiable as required with gradient-based optimization [26]. Conditional *if statements* were smoothed using hyperbolic tangent functions (example in electronic supplementary material). To increase computational speed, we defined muscle–tendon lengths, velocities and moment arms as a polynomial function of joint positions and velocities [27]. We optimized the polynomial coefficients to fit muscle–tendon lengths and moment arms (maximal root mean square deviation: 3 mm; maximal order: ninth) obtained from OpenSim using a wide range of joint positions.

### 2.2. Experimental data

We used experimental data for comparison with simulation outcomes as well as to provide some of the bounds and initial guesses of the predictive simulations. Not all initial guesses were based on experimental data. We collected data (marker coordinates, ground reaction forces and electromyography; recording details in the electronic supplementary material) from one healthy adult. The subject was instructed to walk over the ground at a self-selected speed and to run on a treadmill at 10 km h^−1^. The average walking speed, henceforth referred to as the preferred walking speed, was 1.33 ± 0.06 m s^−1^. We processed the experimental data with OpenSim 3.3 [19]. The musculoskeletal model was scaled to the subject's anthropometry based on marker information from a standing calibration trial. Joint kinematics were calculated based on marker coordinates by applying a Kalman smoothing algorithm [28]. Joint kinetics were calculated based on joint kinematics and ground reaction forces.

### 2.3. Optimal control framework

We formulated predictive simulations of gait as optimal control problems. We identified muscle excitations and gait cycle duration that minimized a cost function subject to constraints describing muscle and skeleton dynamics, imposing left–right symmetry and prescribing gait speed (defined as the distance travelled by the pelvis divided by the gait cycle duration).

This optimal control problem is challenging to solve because of the stiffness of the equations describing muscle and skeleton dynamics. Owing to these stiff differential equations, a small change in muscle excitations can have a large impact on the simulated movement pattern and the cost function because of, for example, the high sensitivity of the ground reaction forces to the kinematics. To overcome this challenge, we used an optimal control method called direct collocation [14,22]. Compared to other methods such as direct shooting [7], direct collocation reduces the sensitivity of the cost function to the optimization variables by reducing the time horizon of the integration. Applying direct collocation results in large sparse nonlinear programming problems (NLP) that readily available NLP solvers can solve efficiently.

We formulated muscle and skeleton dynamics with implicit rather than explicit differential equations, which are more common [24,29]. Using implicit formulations improves the numerical conditioning of the NLP by, for example, removing the need to divide by small muscle activations [24] or invert the mass matrix that is near-singular due to the large range of masses and moments of inertia of the body segments [29]. For the muscle contraction and skeleton dynamics, we introduced additional controls $u_{dF_{t}}$ and $u_{\mathrm{dv}}$ that equal (dynamic constraints) the time derivatives of tendon forces $F_{t}$ and joint velocities *v*, respectively, and we imposed the nonlinear dynamic equations describing muscle contraction and skeleton dynamics as algebraic constraints in their implicit rather than explicit form [24]. We used a slightly different approach for muscle activation dynamics [22]. We introduced additional controls $u_{\mathrm{da}}$ that equal the time derivatives of activations *a* and imposed activation dynamics by linear constraints on *a* and $u_{\mathrm{da}}$. Hence, muscle excitations were eliminated from the problem but can be computed post-processing [22]. Activation dynamics of the ideal actuators driving the arms were described by a linear first-order approximation of a time delay relating excitations $e_{\mathrm{arms}}$ to activations $a_{\mathrm{arms}}$. This equation is linear and continuously differentiable and there was thus no computational rationale for using implicit formulations. Details of the problem formulation are in the electronic supplementary material.

We formulated our problems in MATLAB (The Mathworks Inc., USA) using CasADi [30], applied direct collocation using a third-order Radau quadrature collocation scheme with 50 mesh intervals per half gait cycle and solved the resulting NLP with the solver IPOPT [31]. We increased computational efficiency by applying algorithmic differentiation [30], which is an alternative to finite differences for computing function derivatives required by the NLP solver. In contrast with finite differences, algorithmic differentiation is free of truncation errors. Further, it permits the evaluation of derivatives through both forward and reverse algorithms (the forward algorithm is comparable to finite differences). Typically, the reverse algorithm requires fewer function evaluations than the forward algorithm when the function has many more inputs than outputs, whereas the opposite holds when the function has many more outputs than inputs. Hence, the reverse algorithm is more efficient for computing, for example, the cost function gradient, since the cost function is a single value (one output) that depends on many variables (many inputs). We created custom versions of OpenSim and its dynamics engine Simbody [25] to enable the use of algorithmic differentiation.

### 2.4. Performance criterion underlying healthy human walking

We first sought a performance criterion that could predict healthy human walking by generating simulations, at the subject's preferred walking speed (1.33 m s^−1^), using multi-objective cost functions describing trade-offs between physiologically relevant walking-related performance criteria. Our cost functions included metabolic energy rate, muscle activity, joint accelerations, passive joint torques and arm excitations:

2.1 $$J=\frac{1}{d}\int_{0}^{t_{f}}\left(\underset{\mathrm{Metabolic}\;\mathrm{energy}\;\mathrm{rate}}{\underbrace{w_{1}∥\dot{E}{∥}_{2}^{2}}}+\underset{\mathrm{Muscle}\;\mathrm{activity}}{\underbrace{w_{2}∥a{∥}_{2}^{2}}}+\underset{\mathrm{Joint}\;\mathrm{accelerations}}{\underbrace{w_{3}∥u_{\mathrm{dv},\mathrm{lt}}{∥}_{2}^{2}}}+\underset{\mathrm{Passive}\;\mathrm{torques}}{\underbrace{w_{4}∥T_{p}{∥}_{2}^{2}}}+\underset{\mathrm{Arm}\;\mathrm{excitations}}{\underbrace{w_{5}∥e_{\mathrm{arms}}{∥}_{2}^{2}}}\right)\;dt,$$

where *d* is the distance travelled by the pelvis in the forward direction, $t_{f}$ is half gait cycle duration, $u_{\mathrm{dv},\mathrm{lt}}$ are joint accelerations of the lower limbs and trunk, $T_{p}$ are passive joint torques, *t* is time and $w_{1-5}$ are weight factors. We modelled the metabolic energy rate $\dot{E}$ using a smooth approximation of the phenomenological model described by Bhargava *et al.* [32]. We obtained the parameters for fibre type composition and muscle-specific tension from the literature [33]. We did not include the length dependence of the model's maintenance heat rate. This function is very unsmooth, which is physiologically unlikely and numerically problematic. Further, muscles are working close to their optimal fibre lengths during gait and including length dependency is thus expected to have a minor effect. We smoothed the metabolic energy model in a similar way as the contact model. To avoid singular arcs, situations for which controls are not uniquely defined by the optimality conditions [34], we appended a penalty function *J*_p_ with the remaining controls to the cost function:

2.2 $$J_{p}=\frac{1}{d}w_{u}\int_{0}^{t_{f}}(∥u_{\mathrm{da}}{∥}_{2}^{2}+∥u_{dF_{t}}{∥}_{2}^{2}+∥u_{\mathrm{dv},\mathrm{arms}}{∥}_{2}^{2})\;dt,$$

where $w_{u}=0.001$ and $u_{\mathrm{dv},\mathrm{arms}}$ are joint accelerations of the arms. We explored many sets of weight factors, by manually tuning them, until we found a cost function that predicted human-like walking, henceforth referred to as nominal cost function. We started each optimization from two initial guesses (electronic supplementary material, table S1) and selected the result with the lowest optimal cost. Only one initial guess was based on experimental data.

We investigated the effect of different terms in the cost function by consecutively replacing the metabolic energy rate term by $2w_{1}\sum_{m=1}^{M}{\dot{E}}_{m}$ where *M* is the number of muscles (i.e. not squaring metabolic energy rate), removing the metabolic energy rate term, removing the muscle activity term, lowering the weight on joint accelerations and removing the passive torque term.

### 2.5. Predictive simulations under different conditions

We then tested whether the nominal cost function could predict healthy and pathological gaits when altering gait speed and musculoskeletal properties.

First, we generated predictive simulations at different gait speeds (from 0.73 to 2.73 m s^−1^ by increments of 0.1 m s^−1^). For each speed (except for the preferred walking speed), we used five initial guesses (electronic supplementary material, table S1). Our criterion to evaluate whether the model adopted a walking or running gait was potential and kinetic energy being out-of-phase or in-phase [35].

Second, we investigated the influence of weak hip muscles and ankle plantarflexors during walking. We generated predictive simulations, at the preferred walking speed, while successively decreasing the maximal isometric force of muscles in the corresponding muscle group by 50, 75 and 90%.

Third, we explored the influence of a transtibial passive prosthesis during walking. To model the prosthesis, we removed the ankle and subtalar muscles (including the gastrocnemii) of the right leg and modelled a passive prosthesis by describing ankle and subtalar torques as linear functions of joint angles *q*:

2.3 T=−kq,

where *k* = 800 N m rad^−1^ is torsional stiffness [36]. We reduced the mass of the lower leg and foot segments by 35% and the moment of inertia by 60% compared to the biological leg [36]. We did not alter the foot–ground contact model. To allow for gait asymmetry, we imposed periodicity of the states over a complete gait cycle (except for the pelvis forward position) instead of symmetry over half a gait cycle. We used 100 rather than 50 mesh intervals to account for the longer motion.

### 2.6. Sensitivity analyses

We evaluated the sensitivity of our simulations to different parameters. If not explicitly mentioned, these simulations minimized the nominal cost function at the preferred walking speed. First, we evaluated how using different metabolic energy models, namely the models proposed by Umberger *et al.* [37], Umberger [38] and Uchida *et al.* [33], influenced walking simulations. These models treat negative mechanical work, muscle lengthening heat rate and motor unit recruitment differently (electronic supplementary material, table S2). Second, we tested the influence of increasing the lower bound on muscle activations to simulate co-contraction (using 0.1, 0.15 or 0.2 instead of 0.05). Third, we evaluated the sensitivity of the simulations to the foot–ground contact model parameters. We first calibrated a subset of the contact model parameters (transverse plane locations and radii of the contact spheres) by minimizing tracking errors with respect to the subject's walking data (details in electronic supplementary material). We then used the optimized contact models in predictive simulations*.* Fourth, we evaluated the sensitivity of the results to the number of mesh intervals by using 100 rather than 50 intervals. Finer meshes increase accuracy but also problem size and likely computational time. Finally, we evaluated the sensitivity of the walk-to-run transition speed to the model's peak mechanical power. We increased muscle power by doubling the maximal muscle contraction velocities (from 10 s^−1^ to 20 s^−1^) and generated predictive simulations at increasing gait speeds (from 1.33 to 2.23 m s^−1^ by an increment of 0.1 m s^−1^).

## 3. Results

Our framework generated three-dimensional muscle-driven simulations that converged in an average of 36 min of computational time (over 197 simulations; electronic supplementary material, table S3) on a single core of a standard laptop computer (2.9 GHz Intel Core i7 processor).

**Figure 1.**
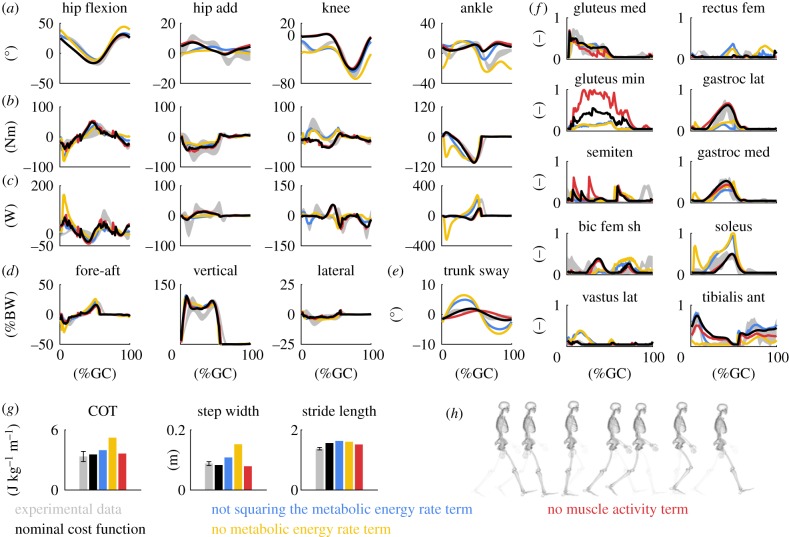
Simulated walking gaits with nominal and alternative cost functions. (*a*) Joint angles (add, adduction). The nominal cost function predicted an extended knee during mid-stance and limited ankle plantarflexion at push-off. Not squaring or removing the metabolic energy rate term from the cost function increased knee flexion but also trunk sway (*e*) and step width (*g*). (*b*) Joint torques. An extended knee resulted in small knee torques but limited ankle plantarflexion did not result in reduced ankle torques. (*c*) Joint powers. Limited ankle plantarflexion resulted in reduced ankle powers. (*d*) Ground reaction forces (BW, body weight; GC, gait cycle). (*e*) Trunk sway (i.e. trunk rotation in frontal plane). (*f*) Muscle activations (gluteus med, gluteus medius; min, minimus; semiten, semitendinosus; bic, biceps; fem, femoris; sh, short head; lat, lateralis; gastroc med, gastrocnemius medialis; ant, anterior). Removing the muscle activity term from the cost function resulted in unrealistically high muscle activations. The experimental electromyography data (grey curves) were normalized to peak nominal activations (black curves). (*g*) Metabolic cost of transport (COT), step width and stride length. The nominal COT matched experimental data [8]. (*h*) Resultant walking pattern with nominal cost function (electronic supplementary material, movie S1). Experimental data are shown as mean ± 2 s.d. (Online version in colour.)

We found that metabolic energy rate, muscle activity, joint accelerations and, to a lesser extent, passive joint torques—all terms squared—were important criteria to capture key features of human walking. We identified a set of weight factors that predicted joint kinematics, kinetics, ground reaction forces and muscle activations resembling experimental data of the subject at the preferred walking speed ($w_{1}=5\;\times 10^{2}/92/\mathrm{body}\;\;\mathrm{mass},w_{2}=2\times 10^{3}/92,w_{3}=5\times 10^{4}/21,w_{4}=1\;\times 10^{3}/15,\;w_{5}=1\times 10^{6}/8$; each weight factor is scaled by the number of elements in the vector from which we take the norm). Minimizing this nominal cost function required 23 min of computational time (electronic supplementary material, table S3) and resulted in a human-like walking gait (figure 1; electronic supplementary material, movie S1). The COT from this simulation, 3.55 J kg^−1^ m^−1^, was in the range of experimental measurements (3.35 ± 0.25 J kg^−1^ m^−1^ [8]) (figure 1). As opposed to previous studies [7–10], we squared the metabolic energy rate term; minimizing energy rate without squaring resulted in exaggerated trunk sway (figure 1; electronic supplementary material, movie S2).

Each term in the proposed nominal cost function was necessary for predicting the prominent features of walking. Removing the metabolic energy rate term resulted in increased trunk sway and step width, whereas removing the muscle activity term resulted in unrealistically high muscle activations for several muscles (figure 1; electronic supplementary material, movie S2). The joint acceleration term was important for convergence of the optimization algorithm and smoothness of the motion, whereas the passive joint torque term limited knee overextension (electronic supplementary material, movie S3).

**Figure 2.**
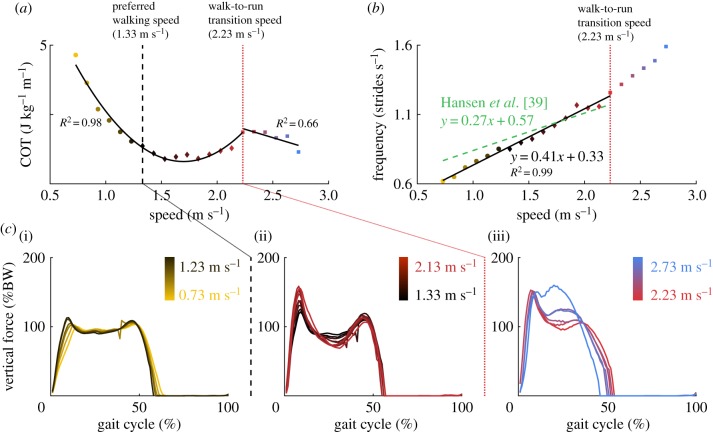
Alterations in gait features with speed. (*a*) Quadratic and linear regressions (black curves) based on simulation results (coloured markers) between metabolic cost of transport (COT) and speed for walking (0.73–2.23 m s^−1^; $R^{2}=0.98$) and running (2.23–2.73 m s^−1^; $R^{2}=0.66$), respectively. (*b*) Linear regression (black curve) based on simulation results from walking (coloured markers) between stride frequency and speed ($R^{2}=0.99$). The regression line is compared with the one obtained from experimental data [39]. (*c*) Vertical ground reaction forces (BW, body weight) at walking speeds less than the preferred walking speed of 1.33 m s^−1^ (i), at walking speeds greater than the preferred walking speed (ii) and at running speeds (iii). Each coloured curve represents a simulation result for a different gait speed. (Online version in colour.)

Our framework predicted a continuum of walking and running gaits as we varied the prescribed gait speed (electronic supplementary material, movie S4). Further, the predicted COT, stride frequency and vertical ground reaction forces changed as a function of speed in agreement with reported data (figure 2). A transition from walking to running occurred at 2.23 m s^−1^ (electronic supplementary material, figure S1). In agreement with the literature, we found quadratic (coefficient of determination $R^{2}=0.98$) and linear ($R^{2}=0.66$) relations between COT and speed for walking and running, respectively (figure 2*a*) [35]; a linear relation ($R^{2}=0.99$) between stride frequency and walking speed (figure 2*b*) [39]; and vertical ground reaction forces whose first peak increased and mid-stance magnitude decreased as walking speed increased (figure 2*c*) [40].

**Figure 3.**
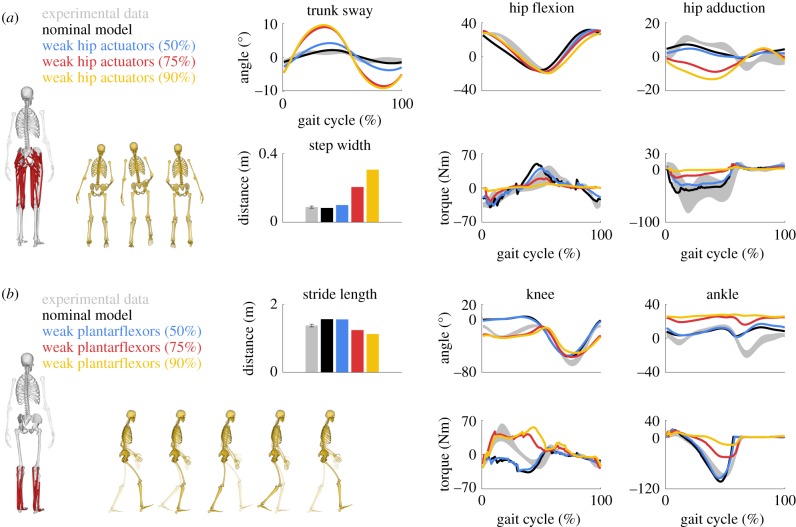
Effect of muscle weakness on walking pattern. (*a*) Hip muscle weakness. Reducing hip muscle strength by 50, 75 and 90% resulted in increased trunk sway and step width and decreased hip torques. (*b*) Ankle plantarflexor weakness. Reducing ankle plantarflexor strength by 50, 75 and 90% resulted in increased knee flexion and ankle dorsiflexion and decreased stride lengths that reduced ankle torques. Experimental data of the healthy subject are shown as mean ± 2 s.d. The simulations minimized the nominal cost function at the subject's preferred walking speed (1.33 m s^−1^). (Online version in colour.)

Altering musculoskeletal properties led to gaits that accurately exhibited clinical gait deficiencies. Reduced hip muscle strength resulted in greater hip circumduction (i.e. conical movement of the legs) to reduce hip torques (figure 3*a*; electronic supplementary material, movie S5). This strategy, known as compensated Trendelenburg gait, may be observed in patients with neural injuries or myopathies affecting hip muscles [41]. Reduced ankle plantarflexor strength resulted in calcaneal gaits that reduced ankle torques (figure 3*b*; electronic supplementary material, movie S6). Such gaits may be observed in children with spastic diplegia who have a weak triceps surae, possibly due to an Achilles tendon lengthening surgery [42].

**Figure 4.**
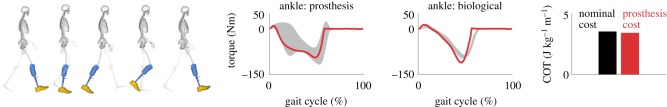
Simulated walking gait of an amputee with a transtibial passive prosthesis. Simulated ankle torques (red curves) matched the average ankle torques of six transtibial amputees [43]. The metabolic cost of transport (COT) for healthy and amputee walking was similar. Experimental data (grey envelopes) are shown as mean ± 2 s.d. The simulations minimized the nominal cost function at an imposed speed of 1.33 m s^−1^. The prosthesis geometry is for visualization only.

Our simulations produced ankle torques and COT that are typical of amputees with a transtibial passive prosthesis. In agreement with experiments [43], ankle plantarflexion torques of the affected leg were larger during early- and mid-stance than torques of the unaffected leg (figure 4; electronic supplementary material, movie S7). The COT was similar to the nominal COT, as expected for physically fit amputees [44].

The sensitivity analyses showed that different metabolic energy models resulted in qualitatively similar walking patterns, although we observed some differences in hip, knee and ankle angles caused by differences in the muscle lengthening heat rate component of the metabolic cost (electronic supplementary material, figure S2, table S2 and movie S8). Increasing the lower bound on muscle activations from 0.05 to 0.1 resulted in larger knee flexion angles, knee torques and vasti activity during stance but also in a larger COT (5.10 J kg^−1^ m^−1^). Lower bounds larger than 0.1 resulted in non-human-like gaits (electronic supplementary material, figure S3). The geometry of foot–ground contact and the mesh density had little influence on the simulations (electronic supplementary material, figures S4–S6). Finally, increasing the model's peak mechanical power reduced the transition speed to 2.13 m s^−1^ (electronic supplementary material, figure S1).

## 4. Discussion

We developed a computationally efficient framework for predictive simulations of three-dimensional human gaits that allowed us to explore a broad range of control strategies and conditions. Our simulations showed that healthy and pathological gaits can emerge from the same underlying control strategy, thereby providing further support for observations based on experimental protocols [45] and conceptual models that did not account for the large redundancy in the musculoskeletal system [5]. In addition, we showed that predictive simulations based on complex three-dimensional musculoskeletal models can capture healthy and pathological human gait mechanics and energetics, a first prerequisite for the use of our models and simulation framework for clinical outcome predictions.

Our framework generated three-dimensional muscle-driven simulations in only 36 min on average, which is more than 20 times faster than existing simulations with similarly complex or simpler models (i.e. between 13 and 60 h) [8–10]. Note that a fair comparison of computational efficiency with published simulations is difficult as none of these studies solved the exact same problems. Further, prior studies might not have prioritized computational efficiency. Our use of direct collocation, implicit differential equations and algorithmic differentiation likely explains the superior computational efficiency of our framework but a comparison with alternative methods is required to further our insight into how each of these components contributed. We also made several assumptions that may have contributed to the rapid convergence of our simulations but that may not always hold. First, imposing symmetry allows simulating only half a gait cycle but is not valid, for example, in impaired gait. Optimizing for a complete gait cycle results in a higher computational time (e.g. prosthesis simulation in electronic supplementary material, table S3). Second, fixing the moving knee flexion axis increases computational speed but might not be valid when studying knee pathologies. Third, using an implicit formulation of activation dynamics eliminates muscle excitations from the optimization problems and, therefore, we used activations instead of excitations to evaluate motor unit recruitment in the metabolic energy model. Similarly, our implicit approach to impose activation dynamics does not allow us to directly formulate constraints on excitations (e.g. imposing that excitations are driven by muscle reflexes).

Our cost function (i.e. control strategy) included both metabolic energy rate and muscle activity, which was important to predict physiological walking. Although human walking is often assumed to result from minimizing only energy consumption [5–8,10], including squared muscle activity might be important for minimizing signal-dependent motor noise since muscle activity is believed to directly affect motor noise [46,47]. Our cost function also included a joint acceleration term that was important to obtain convergence. Minimizing joint accelerations or jerks (i.e. rate of change of joint accelerations) to obtain smooth movements also resulted in good predictions of planar reaching movements [48,49]. However, observed reaching movements were predicted equally well when minimizing uncertainty due to sensorimotor noise [46]. Therefore, it remains unclear whether smoothness of the gait patterns is part of the control strategy or emerges from non-modelled neuro-musculoskeletal features (e.g. robustness against perturbations or soft tissue damping). Minimizing only squared muscle activations led to exaggerated trunk sway and step width (figure 1). Hence, the observation based on two-dimensional models that minimizing muscle activity rather than COT better predicts running kinematics [15] and preferred walking speed in elderly [16] might not hold for simulations based on three-dimensional models.

Our simulations produced walking gaits at low speeds and running gaits at high speeds. Nevertheless, the simulated walk-to-run transition speed (2.23 m s^−1^) was slightly greater than reported values (1.89–2.16 m s^−1^ [50]) and most running gaits presented a longer stance phase than expected (close to 50% of the gait cycle), suggesting that our cost function may not capture all goals during running. Other strategies such as reducing maximum dorsiflexor moment [50] or locomotor variability and avoiding instabilities [51] have been suggested to trigger the walk-to-run transition. Increasing the model's muscle mechanical power reduced the transition speed to 2.13 m s^−1^. This is in agreement with the delayed walk-to-run transition observed in young children that has been attributed to reduced peak mechanical power compared to adults [52]. We might have obtained a similar decrease by reducing the cost of peak mechanical power over mechanical work [52] or by increasing muscle power through increased muscle volumes.

Our simulations produced realistic gaits at different gait speeds, with muscle strength deficits, and with lower leg prosthesis use based on the same control strategy. Hence, a range of healthy and pathological human gaits emerged from the multi-objective cost function that was identified by judging the realism of a simulated gait pattern at a self-selected speed only. Yet this observation does not imply that the proposed cost function represents the underlying physiological control mechanisms that drive locomotion. Although omitting any term in our cost function resulted in less realistic patterns, it is possible that a different set of weight factors or alternative cost functions containing criteria proposed in the literature but not considered in this study (e.g. head stability [53] and angular momentum regulation [54]) will result in equally or more realistic gaits. Especially since many of the proposed criteria are related through system dynamics.

Despite the absence of reflexes and other motor control pathways, our simulations captured the prominent features of human gait. This may be because preferred gait patterns are dictated in part by musculoskeletal mechanics and not only by a control strategy. This would explain why we obtained human-like walking patterns with different cost functions and is in line with observations based on passive walkers that natural dynamics may largely govern locomotion [55]. Second, in a healthy nervous system, different control pathways might interact in a way that optimizes a task-level goal. Our cost function might therefore capture the result of distributed pathways within the central nervous system without explicitly describing these pathways. Such optimization is less likely in the presence of pathologies of the central nervous system, such as spasticity, in which reflex loops are dysregulated. Third, reflexes and other feedback pathways might be especially important to move in a noisy world (i.e. to reject disturbances) but we did not model any noise. This omission might explain the lack of hamstrings activity at terminal swing (semiten in figure 1), which has been reported to be reflex-driven [56]. We expect our predictions to be more accurate if we model known control loops [57], especially in the presence of pathologies or in noisy environments. Alternatively, our framework could be used to test the effect of hypothesized control pathways on human locomotion.

Combining our simulation workflow with experimental studies will advance our understanding of criteria driving gait selection and improve the accuracy of our predictions. The hypothesis that the COT is optimized during gait has been extensively tested both by observing natural behaviour and by manipulating the relation between COT and gait pattern (e.g. [12]). Our simulations suggested that minimizing metabolic rate alone does not result in realistic gaits and pointed to other performance criteria that can be experimentally tested. For example, passive joint torques could be manipulated through braces, whereas manipulating muscle activations or joint accelerations will be more challenging. Our simulations elicited the potential role of the musculoskeletal mechanics, which could also be manipulated experimentally, for example, by locking degrees of freedom, reducing the base of support (cfr., [58]), or adding mass to certain segments. These experiments could be combined with approaches based on inverse optimal control to automate the search for a walking control strategy. To realize the potential of our framework for optimal treatment design, experimental work is also needed to investigate how the control strategy changes in the case of motor control impairments.

We have demonstrated the ability of our simulations to reproduce key features of healthy and pathological human locomotion. Nevertheless, our simulations deviated from measured data in two notable ways. First, our predicted knee flexion during mid-stance was limited, resulting in small knee torques (figure 1). Other predictive studies have reported limited knee flexion during mid-stance and argued that the cause is the lack of stability requirements [14,26]. Similar to these studies, we did not model any stability requirements and included both muscle activity and metabolic rate in the cost function, which might explain why reducing knee torques and, therefore, knee extensor activity was optimal. Co-contraction has been suggested to play a stabilizing role during walking. We found that imposing co-contraction resulted in a more flexed knee during stance at the cost of a higher COT. Future work should investigate more physiologically inspired approaches to account for stability, such as feedback control through spinal reflexes [57]. Alternatively, we could explicitly model and minimize the uncertainty on the simulated movement due to perturbations using approaches from the domain of robust optimal control [59] although such approaches induce significant computational costs. Second, our simulations produced less ankle plantarflexion at push-off (figure 1). The absence of a metatarsophalangeal (MTP) joint might explain reduced plantarflexion, as similar ankle kinematics have been observed experimentally when limiting the range of motion of the MTP joint [60]. The simplistic trunk model might have contributed further to the aforementioned differences between simulated and measured walking patterns. By contrast, the foot–ground contact geometry had little influence on the simulated walking pattern. Our evaluation of the simulated patterns was qualitative rather than quantitative as we found it hard to capture the realism of a gait pattern in a few numbers. Although depending on subjective interpretation, a comprehensive comparison of simulated and measured trajectories along with the animated movements allowed us to judge the realism of our simulations.

Different gaits locally optimize the nominal cost function. We used a discretization scheme with a predefined number of mesh intervals and a gradient-based method to solve the optimization problems. Gradient-based methods find a local optimum, as opposed to the global optimum, and might hence be sensitive to the initial guess. Our simulations based on the nominal cost function at the preferred walking speed converged to similar results whether using an initial guess derived from walking data or a quasi-random initial guess (electronic supplementary material, figure S6), whereas previous studies required initial guesses derived from computationally expensive data-tracking simulations [8,10]. In addition, using a finer mesh led to similar results (electronic supplementary material, figure S6). However, we obtained a different gait pattern when using an initial guess derived from running data (electronic supplementary material, figure S6). The optimal cost and COT of this gait were much larger than those resulting from the two other initial guesses, suggesting different local optima. This is not surprising, since humans adopt a range of different gait patterns at a given speed depending on the context. For example, it has been shown that humans do not always adopt a walking pattern that minimizes energy consumption but prefer this option when instructed to self-explore different patterns [12]. Local optima might hence characterize a model describing human locomotion. How to model the context-dependent selection of a local optimum remains, however, an open question.

Overall, our physics-based computational framework holds the potential to greatly expedite advances in understanding human locomotion. In particular, we expect our efficient simulations, when combined with patient-specific neuro-musculoskeletal models, to enable optimal design of treatments aiming to restore gait function by allowing *in silico* assessment of the effect of changes in the neuro-musculoskeletal system on the gait pattern. Currently, treatment of gait impairments resulting from interactions between motor control and musculoskeletal deficits, such as in cerebral palsy, is often unsuccessful [61]. Optimal treatment design might hence have a large impact on patients' quality of life. Yet the availability of models and methods characterizing these patient-specific deficits is still limited and should be the focus of future research. Further, the design of experimental protocols to collect data required to personalize these models will be particularly important as such protocols should be comprehensive enough to allow for accurate modelling while accounting for practical limitations in clinical contexts. We also envision numerous applications beyond personalized medicine. Our framework can be used to design assistive devices, to simulate gaits of extant and extinct species, to optimize performance in sports by designing equipment and training programmes or to synthesize realistic movements in animations.

## Supplementary Material

Supplementary Material: supporting material, methods, figures, and tables

## Supplementary Material

Movie S1: Predictive simulation of walking with the nominal cost function

## Supplementary Material

Movie S2: Effect of altering the metabolic energy rate and muscle activity terms in the cost function on the predicted walking patterns

## Supplementary Material

Movie S3: Effect of altering the passive joint torque and joint acceleration terms in the cost function on the predicted walking patterns

## Supplementary Material

Movie S4: Predictive simulations of walking and running with the nominal cost function

## Supplementary Material

Movie S5: Effect of weakening the hip muscles on the predicted walking patterns

## Supplementary Material

Movie S6: Effect of weakening the ankle plantarflexors on the predicted walking patterns.

## Supplementary Material

Movie S7: Predictive simulation of walking for an amputee with a transtibial passive prosthesis.

## Supplementary Material

Movie S8: Effect of using different metabolic energy models in the cost function on the predicted walking patterns.

## Data accessibility

All data, code and materials used in this study are available at https://simtk.org/projects/3dpredictsim.
