# Supplementary material for: Rapid predictive simulations with complex musculoskeletal models suggest that diverse healthy and pathological human gaits can emerge from similar control strategies
Source: J R Soc Interface. 2019 Aug 21;16(157):20190402. doi: 10.1098/rsif.2019.0402 (PMC6731507; doi:10.1098/rsif.2019.0402)
Supplement: Supplementary Material: supporting material, methods, figures, and tables [file rsif20190402supp1.pdf]

Supplementary Material for: Rapid predictive simulations with complex musculoskeletal models suggest that diverse healthy and pathological human gaits can emerge from similar control strategies

Antoine Falisse, Gil Serrancolí, Christopher L. Dembia, Joris Gillis, Ilse Jonkers, and Friedl De Groote

Journal of the Royal Society Interface

Experimental data

The subject (female; age: 35 years; height: 170 cm; mass: 62 kg) was instrumented with 45 retro-reflective skin-mounted markers. Three-dimensional marker coordinates were recorded (100 Hz) using a ten-camera motion capture system (Vicon, Oxford, UK). Ground reaction forces and electromyography (EMG) data were recorded (1000 Hz) using two force plates (AMTI, Watertown, USA) and a wireless EMG acquisition system (Cometa Mini Wave, Milano, Italy), respectively. Ground reaction forces were low-pass filtered (6 Hz) using a second-order dual-pass Butterworth filter. EMG data was collected from 14 muscles (peroneus brevis and longus, tibialis anterior, gastrocnemius lateralis and medialis, soleus, vastus lateralis and medialis, adductor longus, rectus femoris, tensor fascia latae, gluteus medius, hamstrings lateralis and medialis) and was processed by band-pass filtering (20-400 Hz), full-wave rectification, and low-pass filtering (10 Hz) using a second-order dual-pass Butterworth filter.

Smooth approximations

We performed smooth approximations of the contact and metabolic energy models to allow having twice continuously differentiable models, which are required when using gradient-based optimization [1]. We performed smooth approximations of conditional *if statements* using hyperbolic tangent functions. For example, the following *if statement*:

$$y = \begin{cases} 0, & x < d \\ a, & x \geq d \end{cases} \tag{1}$$

can be approximated by:

$$f = 0.5 + 0.5 \tanh(b(x - d)), \tag{2}$$

$$y = af, \tag{3}$$

where b is a parameter that determines the smoothness of the transition.

Optimal control problem formulation

We applied direct collocation to solve the optimal control problems underlying the predictive simulations. We sought to find the states, controls, and static (i.e., time-independent) parameters that minimized a cost function subject to dynamic and path constraints.

Optimization variables

States: $\boldsymbol{x}(t)$	Controls: $\boldsymbol{u}(t)$	Static parameter
Muscle activations a<br>Tendon forces F_t<br>Arm activations a_{arms}<br>Joint positions q<br>Joint velocities v	Derivatives of a: u_{da}<br>Derivatives of F_t: u_{dF_t}<br>Arm excitations e_{arms}<br>Derivatives of v (accelerations): u_{dv}	Half gait cycle duration t_f

Cost function

We used multi-objective cost functions describing trade-offs between walking-related performance criteria:

$$J = \frac{1}{d}\int_0^{t_f} \left(\underbrace{w_1 \left\|\dot{E}\right\|_2^2}_{\substack{\text{Metabolic}\\\text{energy rate}}} + \underbrace{w_2 \|a\|_2^2}_{\substack{\text{Muscle}\\\text{activity}}} + \underbrace{w_3 \|u_{dv,lt}\|_2^2}_{\substack{\text{Joint}\\\text{accelerations}}} + \underbrace{w_4 \|T_p\|_2^2}_{\substack{\text{Passive}\\\text{torques}}} + \underbrace{w_5 \|e_{\text{arms}}\|_2^2}_{\substack{\text{Arm}\\\text{excitations}}} \right) dt, \tag{4}$$

where d is distance travelled by the pelvis in the forward direction, $\dot{E}$ is metabolic energy rate (details in main document), $u_{dv,lt}$ are joint accelerations of the lower limbs and trunk, T_p are passive joint torques, t is time, and w_{1-5} are weight factors. To avoid singular arcs, situations for which controls are not uniquely defined by the optimality conditions [2], we also appended a penalty function J_p with the remaining controls to the cost function:

$$J_p = \frac{1}{d}\int_0^{t_f} w_u \left(\|u_{da}\|_2^2 + \|u_{dF_t}\|_2^2 + \|u_{dv,\text{arms}}\|_2^2 \right) dt, \tag{5}$$

where $w_u = 0.001$ weights the penalty function against the other terms in the cost function and $u_{dv,\text{arms}}$ are joint accelerations of the arms.

Dynamic constraints

Due to the implicit formulation of muscle and skeleton dynamics, the dynamic equations were the following:

$$\frac{da}{dt} = s_{da} u_{da}, \tag{6}$$

$$\frac{dF_t}{dt} = s_{dF_t} u_{dF_t}, \tag{7}$$

$$\frac{dq}{dt} = v, \tag{8}$$

$$\frac{dv}{dt} = u_{dv}, \tag{9}$$

$$\frac{da_{\text{arms}}}{dt} = \frac{(e_{\text{arms}} - a_{\text{arms}})}{\tau}, \tag{10}$$

where $s_{da} = 100$ and $s_{dF_t} = 100$ are scale factors, and $\tau = 35$ ms is a time constant. Equations 6-9 correspond to the differential equations for muscle activation, contraction, and skeleton

dynamics. Equation 10 explicitly describes the dynamics of the torque actuators that move the arms and corresponds to a first order approximation of a time delay.

Path constraints

The first set of path constraints defined the implicit muscle (equations 11-13) and skeleton dynamics (equation 14):

$$0 \leq s_{da} u_{da} + \frac{a}{\tau_d}, \tag{11}$$

$$s_{da} u_{da} + \frac{a}{\tau_a} \leq \frac{1}{\tau_a}, \tag{12}$$

$$f_c\left(a, F_t, u_{dF_t}\right) = 0, \tag{13}$$

$$T = f_s\left(q, v, u_{dv}\right), \tag{14}$$

where $\tau_a = 15$ ms and $\tau_d = 60$ ms are muscle activation and deactivation time constants, $f_c(\cdot)$ describes the muscle contraction dynamics according to a Hill-type muscle model [3], and $f_s(\cdot)$ computes net joint torques T according to the skeleton dynamics.

Additional path constraints imposed null pelvis residuals T_{pelvis} (i.e., dynamic consistency; equation 15), that the lower limb and trunk net joint torques equal the sum of muscle, passive, and damping torques (equation 16), and that the arm net joint torques equal the sum of the actuator and damping torques (equation 17):

$$T_{\text{pelvis}} = 0, \tag{15}$$

$$T_{\text{lt}} = \sum_{m=1}^{M} MA_m F_{t,m} + T_p - Bv_{lt}, \tag{16}$$

$$T_{\text{arms}} = a_{\text{arms}} s_{\text{arms}} - Bv_{\text{arms}}, \tag{17}$$

where T_{lt} are torques of the lower limbs and trunk, MA_m is moment arm of muscle m, $B = 0.1$ is a damping factor, T_{arms} are torques of the arms, and $s_{\text{arms}} = 150$ is a scale factor. We imposed left-right symmetry so that we only needed to simulate half a gait cycle. To this aim, we prescribed the relation (i.e., equal or opposite) between the states at the start and end of half a gait cycle (except for the pelvis position in the forward direction). We imposed an average gait speed of $d/t_f = v_{\text{avg}}$, where $v_{\text{avg}} = 1.33$ m s^{-1} was used (subject's preferred walking speed). Finally, to prevent interpenetration of body segments, we imposed minimal distances in the transverse plane between the calcaneus origins (9 cm), between the ipsilateral femur and hand origins (18 cm), and between the tibia origins (11 cm). These distances were informed by experimental data.

Bounds

Joint states and controls (q, v, and u_{dv}) were bounded such that they allowed the generation of healthy and pathological gait patterns. We first used a spline approximation of the experimental walking joint positions $\hat{q}$ to estimate $\hat{v}$ and $\hat{u}_{dv}$. We then computed the minimum ($\hat{q}_{\text{min}}$, $\hat{v}_{\text{min}}$, and $\hat{u}_{dv,\text{min}}$), maximum ($\hat{q}_{\text{max}}$, $\hat{v}_{\text{max}}$, and $\hat{u}_{dv,\text{max}}$) and range ($\hat{q}_r$, $\hat{v}_r$, and $\hat{u}_{dv,r}$) for each of these variables over the gait cycle. Joint states and controls were then bounded as follows:

$$\hat{q}_{\text{min}} - 2\hat{q}_r \leq q \leq \hat{q}_{\text{max}} + 2\hat{q}_r, \tag{18}$$

$$\hat{v}_{\min} - 3\hat{v}_r \leq v \leq \hat{v}_{\max} + 3\hat{v}_r, \tag{19}$$

$$\hat{u}_{dv,\min} - 3\hat{u}_{dv,r} \leq u_{dv} \leq \hat{u}_{dv,\max} + 3\hat{u}_{dv,r}. \tag{20}$$

We manually set the bounds of the pelvis translations:

$$0 \leq q_{\text{pelvis for}} \leq 2, \tag{21}$$

$$0.75 \leq q_{\text{pelvis up}} \leq 1.1, \tag{22}$$

$$-0.1 \leq q_{\text{pelvis lat}} \leq 0.1, \tag{23}$$

where for is forward, up is upward, and lat is lateral. The values are in meters. The bounds of several arm joint angles were also manually set to prevent interpenetration of body segments:

$$0 \leq q_{\text{elbow}}, \tag{24}$$

$$q_{\text{shoulder add}} \leq \hat{q}_{\text{shoulder add,max}}, \tag{25}$$

$$q_{\text{shoulder rot}} \leq \hat{q}_{\text{shoulder rot,max}}, \tag{26}$$

where add is adduction and rot is rotation. Muscle states and controls (a, F_t, u_{da}, u_{dF_t}) were bounded as follows:

$$0.05 \leq a \leq 1, \tag{27}$$

$$0 \leq F_t \leq 5, \tag{28}$$

$$-\frac{\tau_d}{100} \leq u_{da} \leq \frac{\tau_a}{100}, \tag{29}$$

$$-1 \leq u_{dF_t} \leq 1, \tag{30}$$

and arm actuator states and controls (a_{arms} and e_{arms}) were bounded between -1 and 1. We set the lower bound on muscle activations to 0.05 to account for a baseline activation. Since we use an implicit formulation of contraction dynamics, the lower bound is allowed to be 0 but a baseline activation is more physiological.

For the simulations at speeds greater than the preferred walking speed, we adjusted the lower bound of the pelvis pitch and shoulder flexion angles, and the upper bound of the pelvis forward velocity to allow for the generation of running motions:

$$-20^\circ \leq q_{\text{pelvis pitch}}, \tag{31}$$

$$-50^\circ \leq q_{\text{pelvis flex}}, \tag{32}$$

$$\dot{q}_{\text{pelvis for}} \leq 4 \text{ m s}^{-1}, \tag{33}$$

where flex is flexion.

Scaling

To improve the numerical condition of the problem, we scaled the optimization variables so that they had the same order of magnitude [2]. In particular, we scaled joint states and controls, and tendon forces such that their lower and upper bounds were between -1 and 1. To this aim, we used as scale factors the maximum of the absolute value of the lower and upper bounds. Other muscle and arm states and controls were not further scaled, since their lower and upper bounds were already between -1 and 1. Further, as recommended in the literature [2], we scaled the dynamic constraints using the same scale factors as used to scale the states.

Initial guesses

We used two initial guesses (Table S1) for all simulations at the preferred walking speed (1.33 m s^{-1}) and selected the result with the lowest optimal cost. One initial guess (data-informed) relied on experimental data from walking trials for the joint states and controls. The other initial guess (quasi-random) did not rely on any data and described an unrealistic motion. We used the quasi-random initial guess to demonstrate the robustness of our simulations against the initial guess. For the simulations at different speeds, we used five initial guesses (Table S1) and selected the result with the lowest optimal cost. We used more initial guesses for this analysis since we did not have experimental data at each intermediate speed. In all cases, the initial guesses for the muscle states and controls, arm states and controls, and final time were constant across time.

Transcription and solver

We transcribed each optimal control problem into a nonlinear programming problem (NLP) using a third order Radau quadrature collocation scheme with 50 mesh intervals per half gait cycle. We used the interior-point solver IPOPT [4] with the linear algebra package MUMPS [5], using first order derivative information, a limited-memory quasi-Newton method (L-BFGS) to approximate the Hessian of the Lagrangian, and a NLP relative error tolerance of 1×10^{-4} with a maximum of 10,000 iterations. We formulated each optimal control problem in MATLAB (The Mathworks Inc., USA) using CasADi, a tool for nonlinear optimization and algorithmic differentiation [6].

Tracking simulations

We calibrated the contact models while performing muscle-driven tracking simulations of the subject's experimental walking data. We formulated the tracking simulations via direct collocation in the same way as the predictive simulations but we added the transverse plane locations and radii of the contact spheres as optimization variables (static parameters) and used a different cost function:

$$J = \int_{t_i}^{t_f} \Big(w_1 \|q - \hat{q}\|_2^2 + w_2 \Big\| GRF - G\hat{R}F \Big\|_2^2 + w_3 \Big\| GRT - G\hat{R}T \Big\|_2^2 + w_4 \Big\| T - \hat{T} \Big\|_2^2 + w_5 \|a\|_2^2 \Big) \, dt, \quad (34)$$

where t_i and t_f are known initial and final times (t_f was not an optimization variable in this case), $\hat{q}$ are experimental joint positions (except for the upward pelvis position to account for uncertainties in the vertical location of the contact geometries in the foot) obtained from inverse kinematics, $G\hat{R}F$ are experimental ground reaction forces, $G\hat{R}T$ are experimental ground reaction torques expressed in the ground frame, and $\hat{T}$ are experimental net joint torques (except for the pelvis torques that are imposed to be tracked, through path constraints, for dynamic consistency between the tracking simulation and the experimental results from inverse dynamics) obtained from inverse dynamics. We allowed the contact sphere locations and the radii to vary by 25 mm and 50% from their original values [7], respectively. We manually tuned the values of the weight factors, following a heuristic approach, until we found a cost function that well reproduced all experimental data.

Supplementary figures and tables

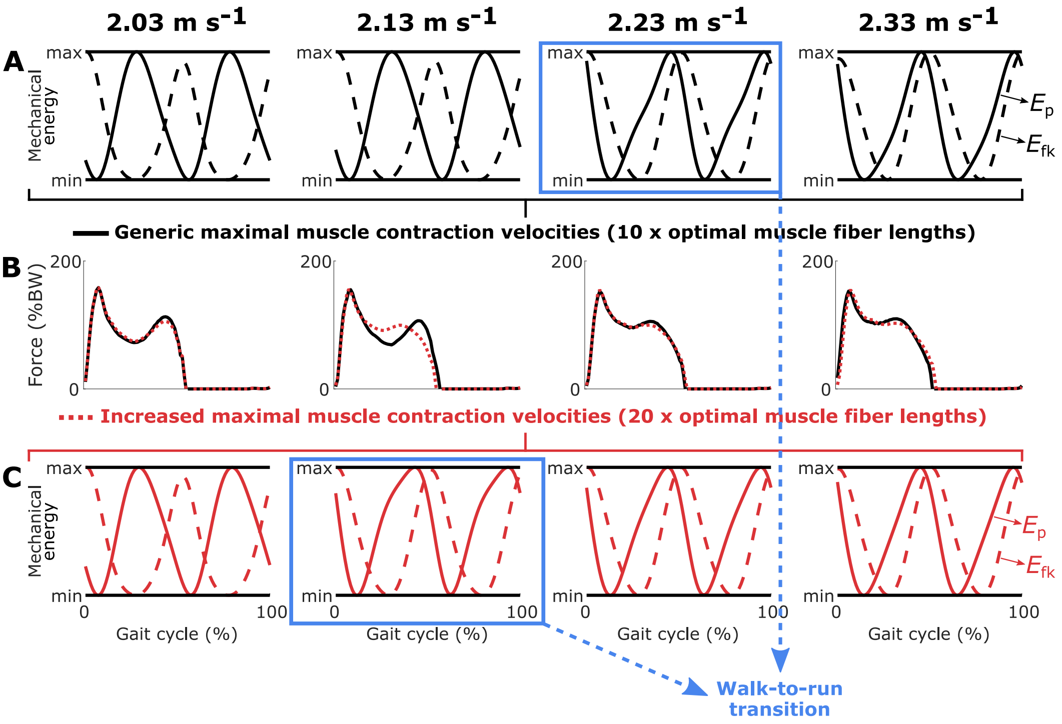

Fig. S1: **Mechanical energy and simulated vertical ground reaction forces around walk-to-run transition speed with and without increased muscle mechanical power.** **(A)** Simulated mechanical energy (gravitational potential energy E_p and forward kinetic energy E_fk) at different speeds with generic maximal muscle contraction velocities. From 2.23 m s^{-1} onwards, E_p and E_fk are in phase (maxima occurring close in time), indicating a transition from walking to running [8]. **(B)** Corresponding simulated vertical ground reaction forces (Force; BW: body weight). Increasing the maximum muscle mechanical power by doubling the maximal muscle contraction velocities had a large influence on the simulated force at 2.13 m s^{-1}. **(C)** Simulated mechanical energy at different speeds with increased maximal muscle contraction velocities. Increasing the maximum muscle mechanical power reduced the walk-to-run transition speed from 2.23 m s^{-1} to 2.13 m s^{-1}. This shift is in agreement with experiments reporting a delayed walk-to-run transition in children due to reduced peak mechanical power as compared to adults [9]. The simulations minimized the nominal cost function.

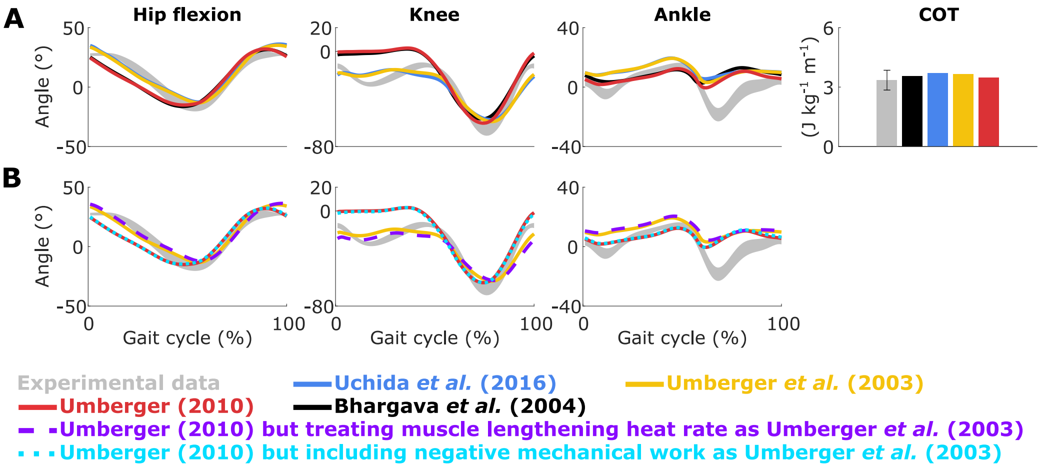

Fig. S2: **Simulated walking gaits with different metabolic energy models. (A)** The models presented by Uchida *et al.* (2016) and Umberger *et al.* (2003) predicted different hip flexion, knee, and ankle angles than the models presented by Bhargava *et al.* (2004) and Umberger (2010). These differences had little influence on the metabolic cost of transport (COT) computed using the model presented by Bhargava *et al.* (2004) based on the simulated states in a post-processing step. Note that there are larger differences in the COT computed with the different metabolic energy models based on the same states. The main differences between the models are the treatment of negative mechanical work and muscle lengthening heat rate (Table S2). **(B)** Treating the muscle lengthening heat rate in Umberger (2010) as in Umberger *et al.* (2003) explained most of the differences between both models. By contrast, including negative mechanical work in Umberger (2010) as in Umberger *et al.* (2003) had little influence on the results. Experimental data is shown as mean ± two standard deviations. The simulations minimized the nominal cost function (except for the use of different metabolic energy models) at the preferred walking speed (1.33 m s^{-1}).

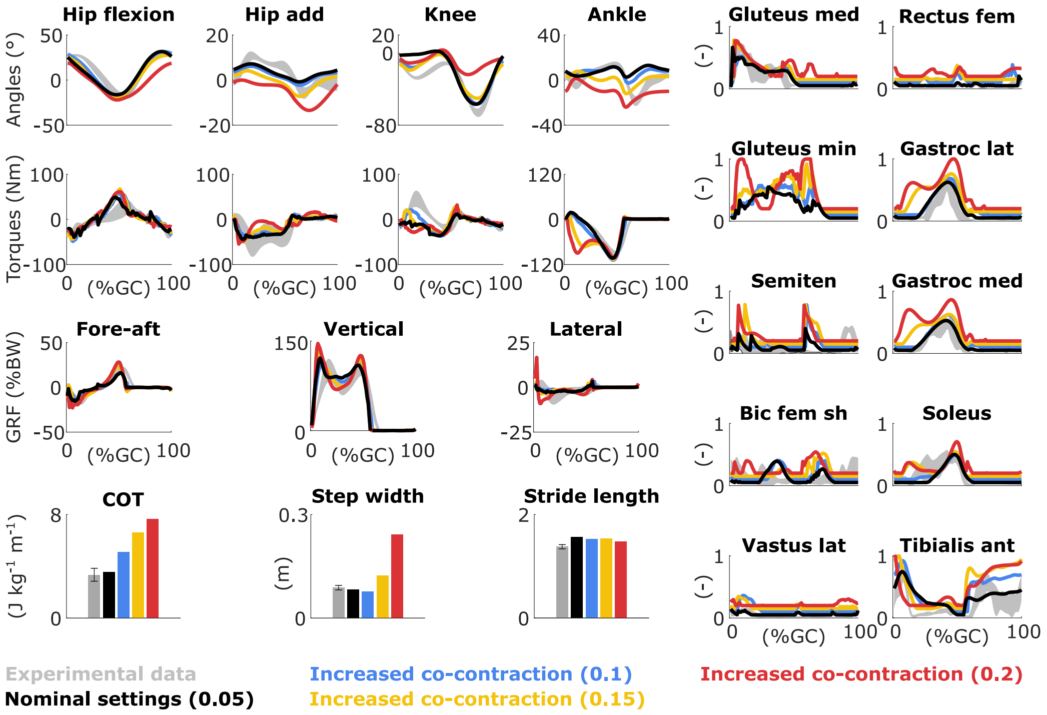

Fig. S3: **Simulated walking gaits with increased co-contraction.** Simulating co-contraction by increasing the lower bounds on muscle activations from 0.05 to 0.1 resulted in larger knee flexion during stance associated with larger knee torques, vasti activity, but also metabolic cost of transport (COT; 5.10 J kg^{-1} m^{-1}). Enforcing more co-contraction (lower bounds on muscle activations equal to 0.15 and 0.2) affected the kinematics, resulting in non-human-like gaits with high COT and muscle activity. Experimental data is shown as mean $\pm$ two standard deviations. The experimental electromyography data (grey curves) was normalized to peak nominal activations (black curves; gluteus med: gluteus medius; min: minimus; semiten: semitendinosus; bic: biceps; fem: femoris; sh: short head; lat: lateralis; gastroc med: gastrocnemius medialis; ant: anterior). GRF: ground reaction forces; BW: body weight; GC: gait cycle; add: adduction.

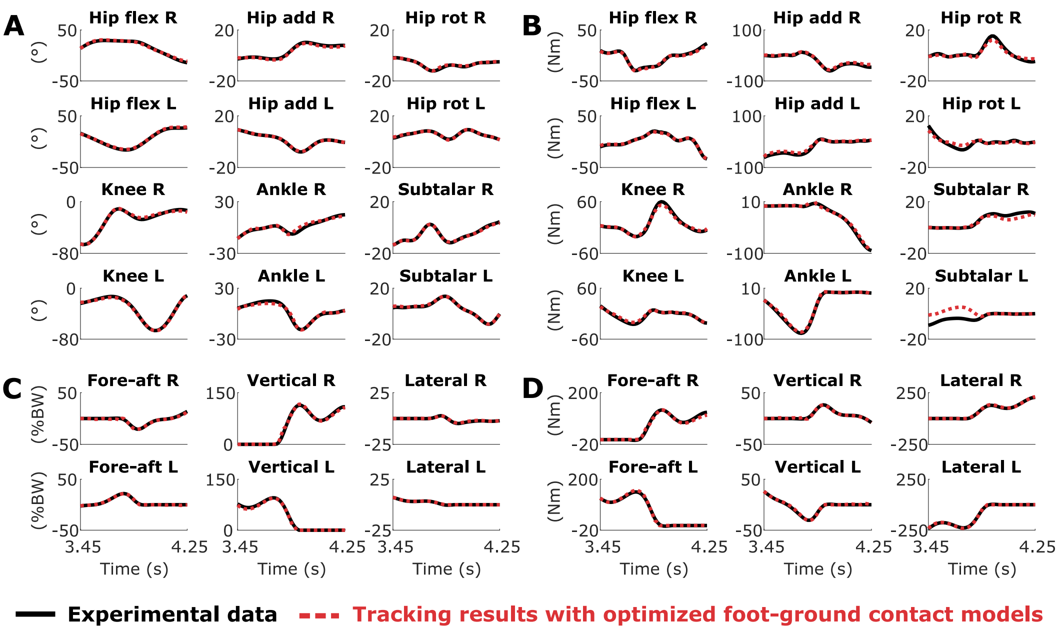

Fig. S4: **Tracking simulations with optimized foot-ground contact models.** The musculoskeletal model with optimized contact models (optimized contact sphere locations and radii) accurately reproduced experimental **(A)** joint angles (flex: flexion; add: adduction; rot: rotation; R: right; L: left), **(B)** joint torques, **(C)** ground reaction forces (BW: body weight), and **(D)** ground reaction torques. Ground reaction forces and torques are expressed in the ground frame. This indicates that the musculoskeletal model is sufficiently complex to reproduce experimental walking data.

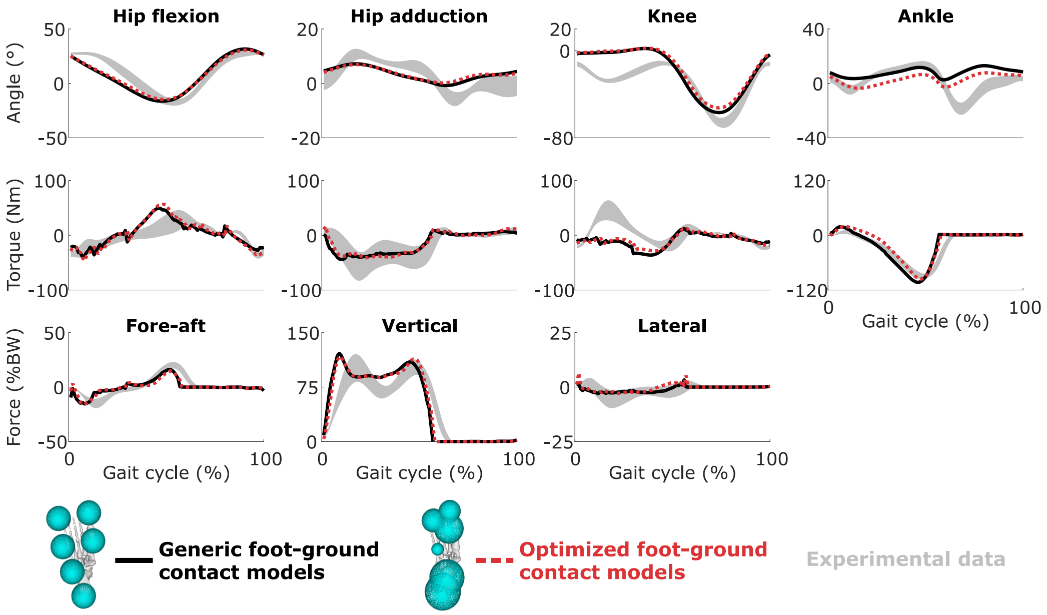

Fig. S5: **Simulated walking gait with optimized foot-ground contact models.** Despite large differences in contact geometry, the use of optimized contact models instead of generic models from the literature [7] had little influence on the predicted gait pattern (Force: ground reaction force; BW: body weight), except for a small offset in the ankle angle. This suggests a low sensitivity of the results to the contact models. The foot diagrams depict a down-up view of the configuration of the contact spheres of the right foot. Experimental data is shown as mean ± two standard deviations. The simulations minimized the nominal cost function at the preferred walking speed (1.33 m s^{-1}).

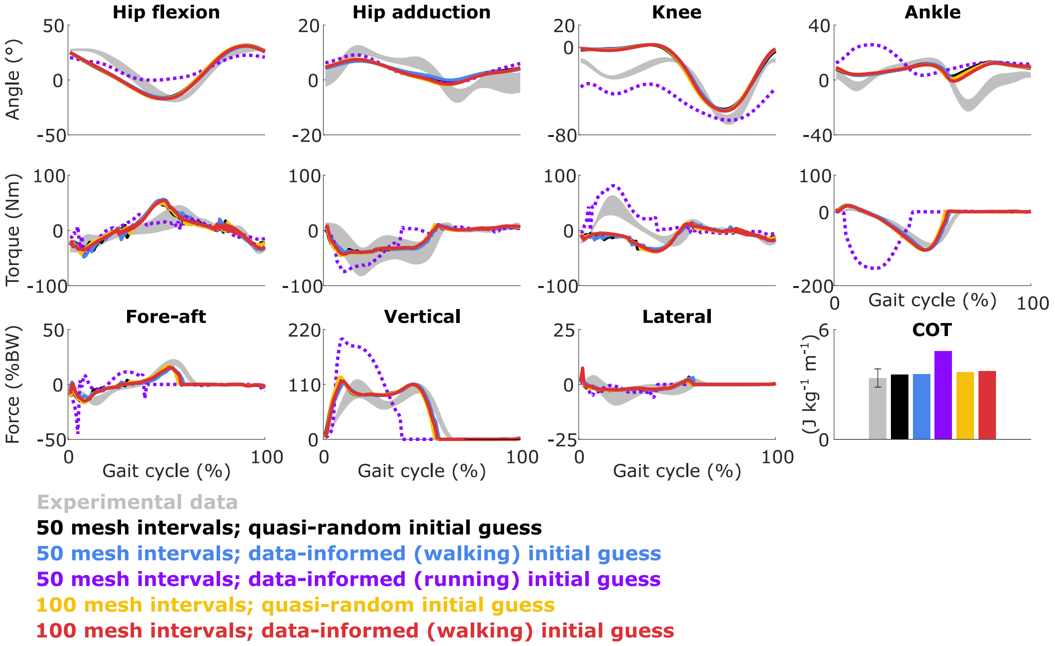

Fig. S6: **Simulated walking gaits with data-informed (walking and running) and quasi-random initial guesses, and with finer mesh.** Varying the initial guess (between data-informed (walking) and quasi-random) and increasing the number of mesh intervals (from 50 to 100) had, overall, little influence on the predicted gait patterns (Force: ground reaction force; BW: body weight; COT: metabolic cost of transport). The four curves nearly coincide. By contrast, using the initial guess derived from running data led to the prediction of a different gait pattern. Yet that simulation had a much larger optimal cost than the simulations resulting from the two other initial guesses with 50 meshes, meaning that the optimal control problems converged to different local optima. Similar to humans, the model can hence adopt different gaits at the same speed. The quasi-random initial guess resulted in a lower optimal cost with both meshes as compared to the data-informed initial guesses. A finer mesh resulted in smoother control and state trajectories as can be appreciated when looking at the hip flexion torques. Computational times increased when increasing the number of mesh intervals from 50 (25, 21, and 53 minutes with the quasi-random, data-informed (walking), and data-informed (running) initial guesses, respectively) to 100 (62 and 69 minutes with the quasi-random and data-informed initial guesses, respectively). Experimental data is shown as mean ± two standard deviations. The simulations minimized the nominal cost function at the preferred walking speed (1.33 m s^{-1}).

States $\boldsymbol{x}$ / Controls $\boldsymbol{u}$ Static parameters $\boldsymbol{p}$		Joints		Muscles and Arms			Time
		$\boldsymbol{x}$	$\boldsymbol{u}$	$\boldsymbol{x}$	$\boldsymbol{u}$		$\boldsymbol{p}$
		q	v / u_{dv}	a, F_t, a_{arms}	u_{da}, u_{dF_t}	e_{arms}	t_f
**Preferred walking speed**	**Data-informed walking**	$\hat{q}_{\text{walk}}$	Spline-based derivatives of $\hat{q}_{\text{walk}}$	0.1	0.01	0.1	0.6
	**Quasi-random**	0 except $q_{\text{pelvis}} = f_q(v_{\text{avg}}, t_f)$ $q_{\text{pelvis up}} = \text{standing}$	0 except $v_{\text{pelvis for}} = v_{\text{avg}}$	0.1	0.01	0.1	0.6
**Different speeds**	**Data-informed (walking)**	$\hat{q}_{\text{walk}}$	Spline-based derivatives of $\hat{q}_{\text{walk}}$	0.1	0.01	0.1	$f_t(v_{\text{avg}})$
	**Data-informed (running)**	$\hat{q}_{\text{run}}$	Spline-based derivatives of $\hat{q}_{\text{walk}}$	0.1	0.01	0.1	$f_t(v_{\text{avg}})$
	**Quasi-random**	0 except $q_{\text{pelvis}} = f_q(v_{\text{avg}}, t_f)$ $q_{\text{pelvis up}} = \text{standing}$	0 except $v_{\text{pelvis for}} = v_{\text{avg}}$	0.1	0.01	0.1	$f_t(v_{\text{avg}})$
	**Full optimal solution (OS) at closest speed**	OS at closest speed					
	**Partial optimal solution (OS) at closest speed**	OS at closest speed (q_{opt})	Spline-based derivatives of (q_{opt})	0.1	0.01	0.1	OS at closest speed

Table S1: **Details of the initial guesses.** $\hat{q}_{\text{walk}}$ and $\hat{q}_{\text{run}}$ refer to the walking and running joint positions, respectively, averaged across experimental trials, pelvis for and pelvis up refer to the forward and upward pelvis translations, respectively, $f_q(v_{\text{avg}}, t_f)$ describes a linear displacement as a function of speed v_{avg} and time t_f, standing refers to the upright standing posture and $f_t(v_{\text{avg}})$ is an expression for t_f as a function of v_{avg} (t_f from 0.70 to 0.35 s by increments of -0.018 s associated with imposed speed v_{avg} from 0.73 to 2.73 m s^{-1} by increments of 0.1 m s^{-1}). For the initial guesses based on the full and partial optimal solutions at closest speed, we started from the preferred walking speed (1.33 m s^{-1}) for which we selected the result with the lowest optimal cost from the data-informed (walking and running) and quasi-random initial guesses. For the remaining speeds, optimal results at the closest speed used to generate the initial guess were obtained using the same approach for determining the initial guess.

**Metabolic energy model**	**Negative mechanical work**	**Muscle lengthening heat rate**	**Motor unit recruitment model**
**Bhargava *et al.* (2004)**	Excluded	$f_B(\cdot)$	Included
**Umberger *et al.* (2003)**	Included	$f_U(\cdot)$	Excluded
**Umberger (2010)**	Excluded	$\frac{3}{40} f_U(\cdot)$	Excluded
**Uchida *et al.* (2016)**	Included	$f_U(\cdot)$	Included

Table S2: **Main differences between metabolic energy models.** The models of Umberger *et al.* (2003), Umberger (2010), and Uchida *et al.* (2016) are the same except in their treatment of negative mechanical work, muscle lengthening heat rate, and motor unit recruitment. The functions $f_B(\cdot)$ and $f_U(\cdot)$ describe the muscle lengthening heat rate for the models of Bhargava *et al.* (2004) and Umberger *et al.* (2003), respectively. In Umberger (2010), $f_U(\cdot)$ is scaled down with respect to Umberger *et al.* (2003) to compensate for the exclusion of negative mechanical work.

<table>
<tr><th>N_m</th><th>Speed</th><th colspan="2">Case</th><th>N_s</th><th colspan="3">Computational time
mean ± std (minute)</th></tr>
<tr><td rowspan="13">50</td><td rowspan="11">Preferred walking speed</td><td colspan="2">Nominal cost function</td><td>2</td><td>23 ± 2</td><td rowspan="13">35 ± 14</td><td rowspan="15">Total
36 ± 15

IPOPT
25 ± 11

NLP
10 ± 4</td></tr>
<tr><td rowspan="5">Alternative cost functions</td><td>No metabolic energy rate term</td><td>2</td><td>29 ± 7</td></tr>
<tr><td>No muscle activity term</td><td>2</td><td>38 ± 2</td></tr>
<tr><td>No squaring metabolic energy rate term</td><td>2</td><td>31 ± 2</td></tr>
<tr><td>No passive joint torque term</td><td>2</td><td>25 ± 3</td></tr>
<tr><td>Lower weight on joint accelerations</td><td>2</td><td>51 ± 10</td></tr>
<tr><td rowspan="2">Muscle strength deficits</td><td>Weak hip muscles</td><td>*6</td><td>30 ± 5</td></tr>
<tr><td>Weak ankle plantarflexors</td><td>*6</td><td>32 ± 5</td></tr>
<tr><td rowspan="3">Sensitivity analyses</td><td>Metabolic energy models</td><td>10</td><td>44 ± 11</td></tr>
<tr><td>Co-contraction</td><td>*6</td><td>35 ± 19</td></tr>
<tr><td>Contact models</td><td>2</td><td>26 ± 3</td></tr>
<tr><td rowspan="2">Different speeds</td><td colspan="2">Nominal cost function:
from 0.73 to 2.73 m s^{-1}</td><td>$99</td><td>35 ± 14</td></tr>
<tr><td colspan="2">Increased maximal muscle contraction velocities:
from 1.33 to 2.23 m s^{-1}</td><td>¶52</td><td>35 ± 17</td></tr>
<tr><td rowspan="2">100</td><td rowspan="2">Preferred walking speed</td><td colspan="2">Nominal cost function</td><td>2</td><td>65 ± 6</td><td rowspan="2">71 ± 24</td></tr>
<tr><td colspan="2">Passive transtibial prosthesis</td><td>2</td><td>77 ± 40</td></tr>
</table>

Table S3: **Details of computational times for the 197 simulations.** N_m and N_s are for number of mesh intervals and simulations, respectively. *6 results from simulations with three levels of weakness (50, 75, and 90%) or co-contraction (0.1, 0.15, and 0.2) with two initial guesses. $99 results from simulations at 20 different speeds (from 0.73 to 2.73 m s^{-1} by increments of 0.1 m s^{-1} except for the preferred walking speed) with five initial guesses plus the preferred walking speed with the data-informed (running) initial guess minus two simulations that we excluded (no optimal solution). The simulations at the preferred walking speed with the data-informed (walking) and quasi-random initial guesses are counted in the nominal cost function case (first row). ¶52 results from simulations at 10 different speeds with increased maximal muscle contraction velocities (from 1.43 to 2.33 m s^{-1} by increments of 0.1 m s^{-1}) with five initial guesses plus the preferred walking speed with the data-informed (walking and running) and quasi-random initial guesses minus one simulation that we excluded (no optimal solution). In the last row, we distinguish the time spent in IPOPT and to evaluate the NLP functions from the total time.

References

[1] A. D. Koelewijn, E. Dorschky, and A. J. van den Bogert, "A metabolic energy expenditure model with a continuous first derivative and its application to predictive simulations of gait," *Computer Methods in Biomechanics and Biomedical Engineering*, vol. 21, no. 8, pp. 521–531, 2018.

[2] J. T. Betts, "The optimal control problem," in *Practical Methods for Optimal Control and Estimation Using Nonlinear Programming*, pp. 123–218, Philadelphia: SIAM, 2 ed., 2010.

[3] F. De Groote, A. Kinney, A. Rao, and B. Fregly, "Evaluation of direct collocation optimal control problem formulations for solving the muscle redundancy problem," *Annals of Biomedical Engineering*, vol. 44, no. 10, pp. 2922–2936, 2016.

[4] A. Wächter and L. T. Biegler, "On the implementation of an interior-point filter line-search algorithm for large-scale nonlinear programming," *Mathematical Programming*, vol. 106, no. 1, pp. 25–57, 2006.

[5] P. R. Amestoy, I. Duff, and J.-Y. L'Excellent, "Multifrontal parallel distributed symmetric and unsymmetric solvers," *Computer Methods in Applied Mechanics and Engineering*, vol. 184, no. 2-4, pp. 501–520, 2000.

[6] J. A. E. Andersson, J. Gillis, G. Horn, J. B. Rawlings, and M. Diehl, "CasADi : a software framework for nonlinear optimization and optimal control," *Mathematical Programming Computation*, vol. 11, no. 1, pp. 1–36, 2019.

[7] Y.-C. Lin and M. G. Pandy, "Three-dimensional data-tracking dynamic optimization simulations of human locomotion generated by direct collocation," *Journal of Biomechanics*, vol. 59, pp. 1–8, 2017.

[8] D. M. Bramble and D. E. Lieberman, "Endurance running and the evolution of Homo," *Nature*, vol. 432, no. 7015, pp. 345–352, 2004.

[9] J. R. Usherwood, T. Y. Hubel, B. J. H. Smith, Z. Self Davies, and G. Sobota, "The scaling or ontogeny of human gait kinetics and walk-run transition: the implications of work vs. peak power minimization," *Journal of Biomechanics*, vol. 81, pp. 12–21, 2018.
